# The new silicone elastometric half-piece respirator, VJR-NMU: A novel and effective tool to prevent COVID-19

**DOI:** 10.1371/journal.pone.0237206

**Published:** 2020-12-31

**Authors:** Anan Manomaipiboon, Sujaree Pupipatpab, Pongsathorn Chomdee, Anusang Chitsomkasem, Yutthana Apichatbutr, Pathiporn Boonyapatkul, Thananda Trakarnvanich

**Affiliations:** 1 Faculty of Medicine, Vajira Hospital, Navamindradhiraj University, Bangkok, Thailand; 2 Urban Community Development, Navamindradhiraj University, Bangkok, Thailand; Universitat de Valencia, SPAIN

## Abstract

Filter facepiece respirators (FFRs) are critical for preventing the transmission of respiratory tract infection disease, especially the dreadful coronavirus 2 (SARs-CoV-2). The N95 mask is a prototype, high-efficiency protective device that can effectively protect against airborne pathogens of less than 0.3 μm. The N95 mask is tightly fitting and has high filtration capacity. The ongoing COVID-19 pandemic has led to a greater requirement for FFR. This rising demand greatly exceeds current production capabilities and stockpiles, resulting in shortages. To address this, our team has invented a new type of half-piece respirator made from silicone and assembled with HEPA or elastostatic filter. A variety of methods have been used to evaluate this new device, including a qualitative fit test with the Bitrex^®^ test kit and filtration test. The preliminary results showed that the new elastometric respirators pass the fit test. The filtration tests also confirmed the superiority of the new respirator over traditional N95 masks, with a mean performance of protection greater than 95%. For the filters, we used two types: SafeStar, which is a kind of HEPA filter; and CareStar, which is considered an elastostatic filler. CareStar was developed to filter virus and bacteria in the operating room, with a limit duration of use up to 24 h, while the safe star was designed for 72 h use and has the quality equivalent to a HEPA filter. Our study demonstrated superior filtration efficacy of both filters, more than 98% even after 24 h of use. CareStar has significantly more filtration efficacy than a safe star (p < 0.001). In conclusion, the development of our new N99 half-piece respirator should ultimately be applicable to healthcare workers with at least non-inferiority to the previously used N 95 respirators. As a universal masking policy is generally implemented, health care workers who are at risk must be protected with appropriate devices. Currently, the adequate supply of such equipment is not feasible. The advent of the new protective device will help protect healthcare workers and replenish the shortage of N95 respirators during the COVID-19 pandemic.

## Introduction

Since the rapid spread of SARs-CoV-2 worldwide, resulting in the novel coronavirus disease 2019 (COVID-19) pandemic, the shortage of personal protective equipment (PPE), including surgical masks and N95 respirators, has been a serious concern [1]. The transmission of SARs-CoV-2 can occur by contact or droplets released from infected persons when coughing, talking, and sneezing [2]. Airborne transmission might occur during aerosol-generating procedures, such as tracheal intubation [3]. Therefore, health care personnel (HCP), especially frontline workers, should be properly protected with appropriate equipment. A filtering facepiece and N95 respirators are used as high-performance filtering masks to protect staff against both droplets and aerosols [4]. To avoid cross contamination, these devices are designed to be disposable after a single use [5]. Consequently, the consumption of FFP masks has been overwhelmed, and a supply shortage for HCP has already been reported in many countries [6].

**Fig 1:**
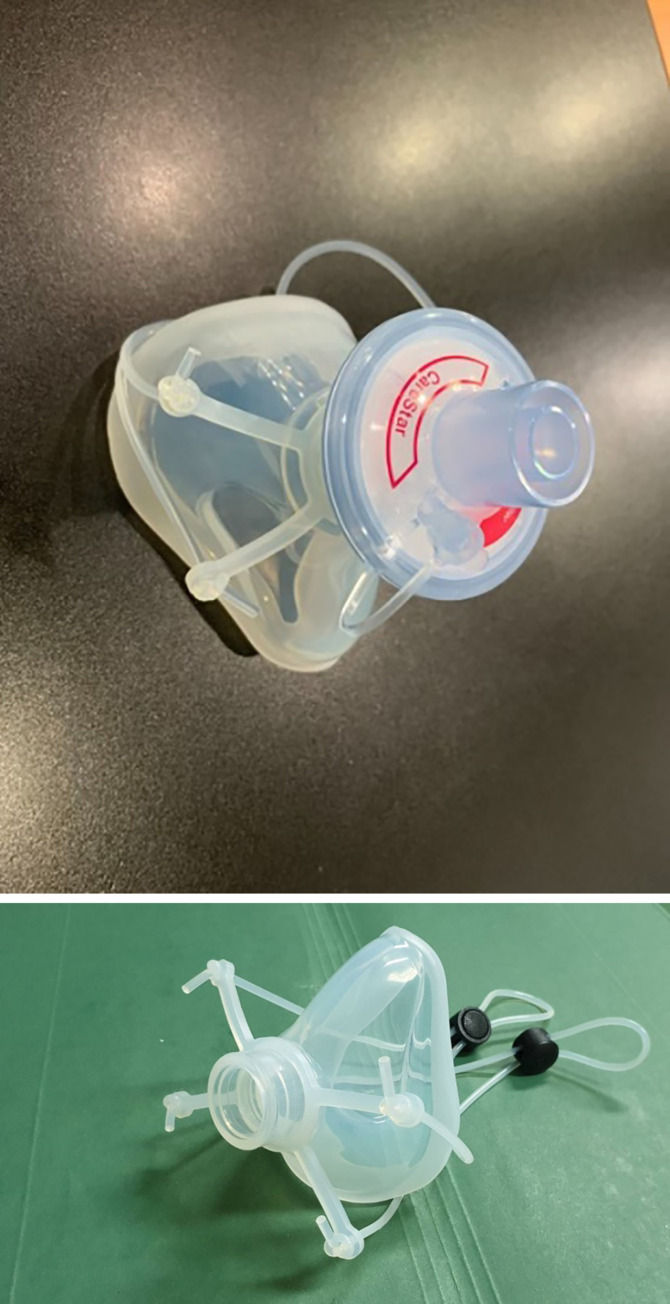
Silicone elastometric half- piece respirator.

To meet the need for FFRs during a pandemic, Navamindradhiraj University has invented a new model of silicone N99 facepiece respirator by using the silicone mask and the HEPA filter normally used in the operating room (CareStar or SafeStar, Draeger, Germany) (Fig 1). This filter has a minimum filtration efficiency of ≥ 99% to the challenge aerosols consisting of particles in the most penetrating particle size range (approximately 0.3 μm). This can prevent the penetration of viruses and bacteria and improves on the protective efficacy of the traditionally used N95 respirators. Here we describe a novel silicone N95 respirator aimed at HCPs who are in direct contact with patients, and capable of contributing to the replenishment of N95 masks.

**Fig 2:**
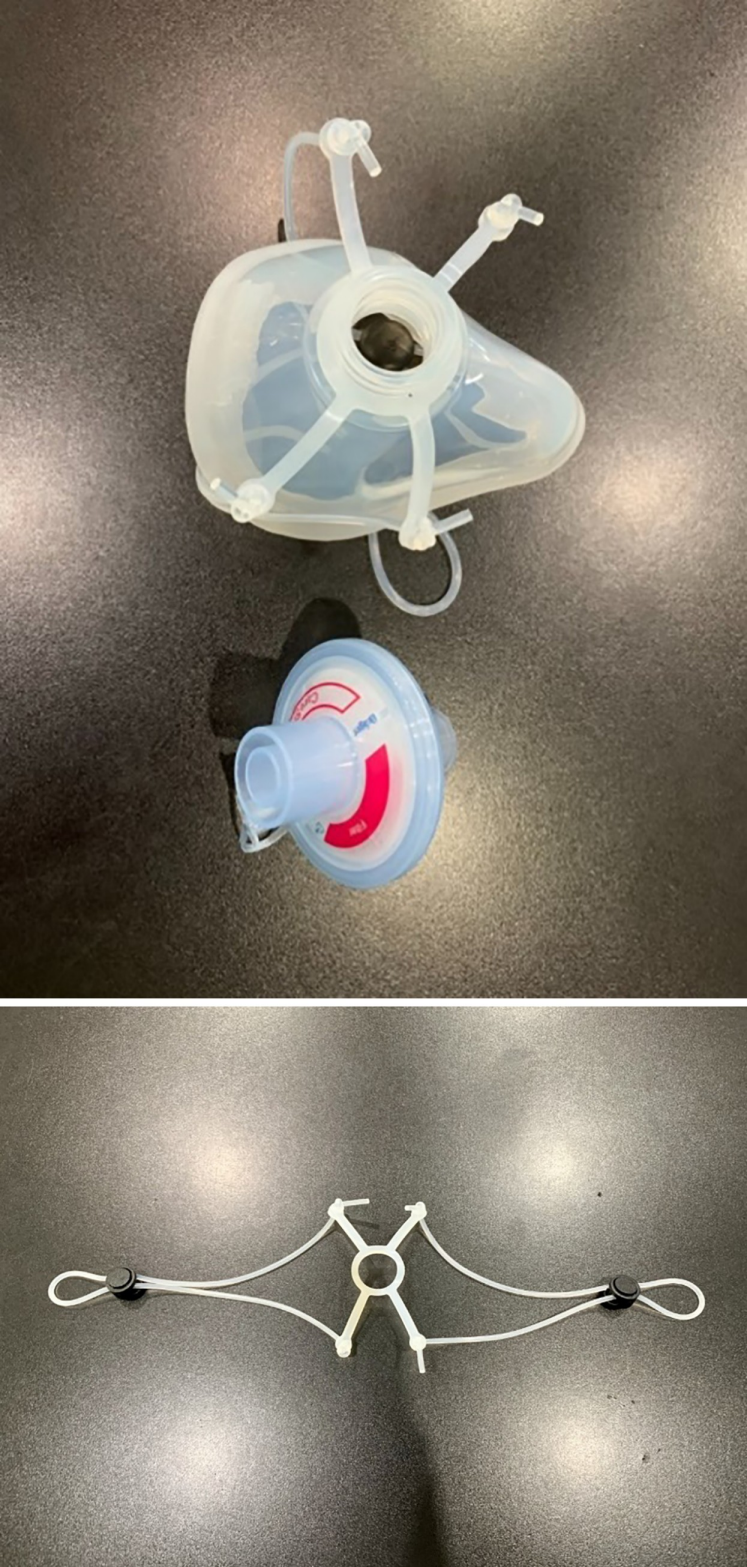
Components of the silicone mask. The respirator consists of a silicone mask and filter.

An important part of the silicone mask is the O-ring strap which can be adjusted to prevent face-seal leakage (Fig 2).

### Components of the N99 mask

**Fig 3:**
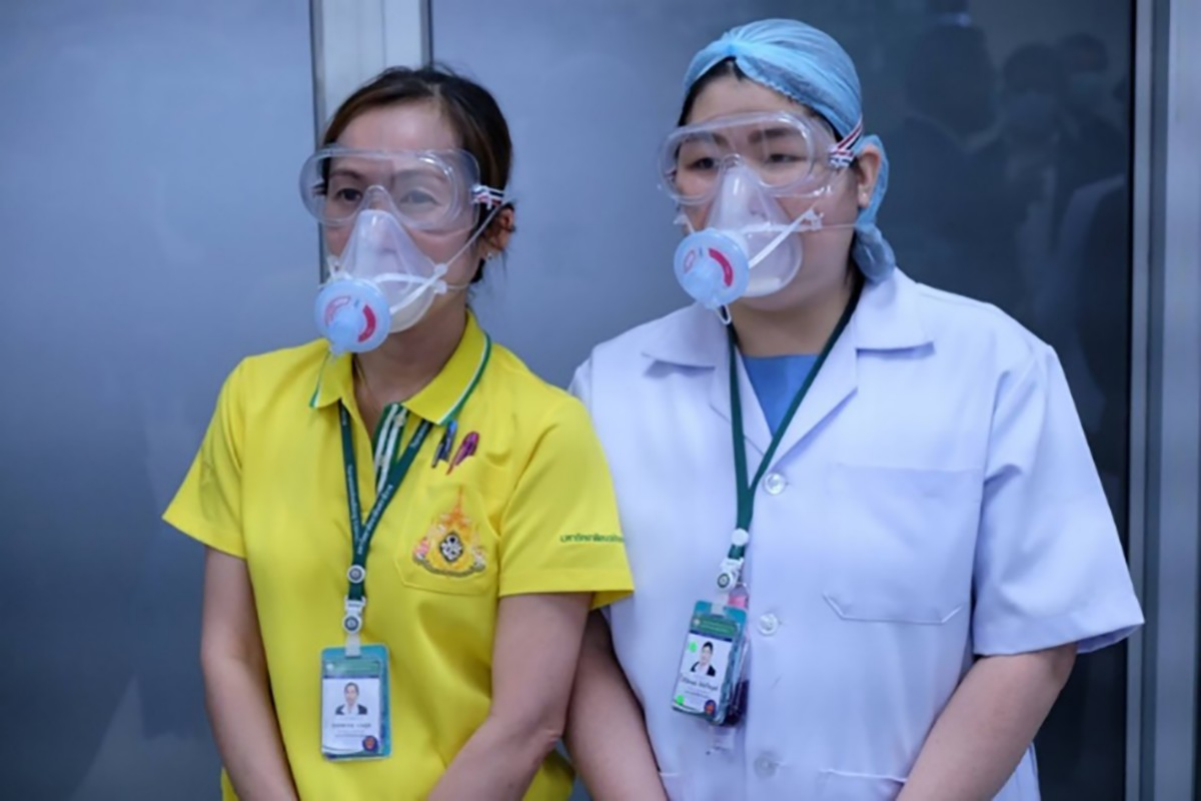
Health care workers wearing silicone elastometric half-piece respirator (with permission from the personnels).

The silicone elastometric mask is made using Silibione MM Series 71791 U silicone (Bluestar silicones, Shanghai, China), which are elastomers comprising of polymethyl vinyl siloxane gums and silica. In this particular series, these silicone rubbers are cured after the addition of a vulcanizing agent (chosen as a function of the production process). The heat cure is done after the addition of an organic peroxide compound and post-curing at 200°C after vulcanization. This series includes four products that differ by their hardness once cured: 40, 50, 60, and 70 types. Once processed, the Silibione MM series 71791 U are intended for food contact and biomedical applications. The advantages of the Silibione MM series 71791 U include easy processing, highly transparent, and excellent mechanical properties (including high tear strength and a good compromise between tear strength and compression set). The Silibione MM series 71791 U is also very resistant to oxidizing agents for all sterilization modes, and is chemically inert. Moreover, samples of the silicone MM series have been subjected to migration heats in accordance with European and American regulations (https://silicones.elkem.com/EN/our_offer/Product/90000717/_/SILBIONE-MM-71791-60-U). The masks are made in three sizes: small, medium, and large. We assembled the silicone with a HEPA filter (CareStar^®^ electrostatic filter), which is normally used together with a ventilator to filter bacteria and viruses from airborne transmission (Fig 2). The efficiency of the silicone mask is based on the efficiency of the air filter, which is considered a high-performing electrostatic filtration medium (Fig 3).

As required by Occupational Safety and Health Administration OSHA) [7], the half piece respirators need to pass the fit test to identify those individuals who do not achieve a sufficiently good fit necessary for adequate protection. The performance of this respirator was also determined by measuring percent leakage under constant airflow [8]. The purposes of this study were to evaluate the fitting characteristic of our newly invented silicone elastometric respirator and the performance against 30-μm particles using NaCl aerosols. The study was conducted to achieve two specific research objectives: (1) to investigate the fit characteristics of the novel silicone mask and whether the strap adjustment can help reduce the face-seal leakage; and (2) to determine the level of performance by measuring the inward leakage of the generated aerosols with a new filter and a used filter (for up to 24 h).

## Materials and methods

### Subjects

Participants were healthcare workers at Vajira Hospital, Bangkok, Thailand. The Vajira Institutional Review Board, Faculty of Medicine, Vajira Hospital, Navamindradhiraj University, approved the protocol, and all participants provided written informed consent. The individuals in this manuscript have given written informed consents to publish these case details. The inclusion criteria were: healthy volunteers, 18 to 60 years old. The major exclusion criteria were: contraindications to fit tests, such as asthma, congestive heart failure, anosmia, and ageusia. Forty-three people (22 males, 21 females; mean age 28.71 ± 7.04 years) participated in this study. We excluded one male due to test intolerance (difficulty in breathing while putting on the hood cover). The other female was excluded because of a failed sensitivity test (no tests after 30 squeezes of sensitivity test solution). The remaining 41 people (21 males and 20 females) chose the proper size of silicone respirators to perform the qualitative fit testing.

A previous study by Zhuang et al. [9], who developed respirator fit test panels by the bivariate distribution of face length and lip length in 3,997 of respirator users demonstrated that 95% of the subjects were included in the panel. This was done to ensure that the samples selected were accurately representative of respirator users in the US We also performed a bivariate analysis and found that 65% were in the boundary. The number was quite low and could be due to the small sample size. (data in the S1 File)

### Filtering facepieces

The newly invented silicone half-piece respirators were tested. The configuration and model have been described above. There are three available sizes: small, medium, and large. None of the models tested in this study had exhalation valves. The CareStar filter is an electrostatic filter, and SafeStar is a HEPA filter product that is high performance, retaining at least 99.99% of bacterial and virus, with high hydrophobicity and transparent housing for visual control.

### The fit test procedure

A fit test was done with qualitative (Bittrex Solution aerosol, Qualitative Fit Test). The protocol was conducted in accordance with the protocol from the OSHA respiratory protection standard [10], including the number, type, and duration of the exercise, and the seal checks in accordance with the manufacturer’s instructions [11].

### Bitrex fit test

The Bittrex test uses a person’s ability to test a bitter solution to determine whether a respirator fits properly [12]. Each subject was given a taste-threshold screening test prior to each fit test to ensure that he or she could taste. This process was done without the subject wearing a respirator. Inject ten (10) squeezes of the test solution aerosol into the hood through the hole in front of the enclosure and ask the subject if he can detect the bitter taste of Bitrex. If the subject does not detect bitter taste, inject an additional ten squeezes into the hood. If the subject still does not detect bitter taste, inject an additional ten squeezes, for a total of 30 squeezes. If 30 squeezes were inadequate to elicit a response from the subject, this subject could not be fit tested with the Bitrex test. If the subject could detect bitter taste, the number of squeezes required to produce a taste response should be noted, i.e., 10, 20 or 30 squeezes, even if the subject tasted the Bitrex on a number of squeezes other than multiples of ten.

After passing the sensitivity test, the subject will proceed through the seven steps of the fit test, as follows: breathe normally; breathe deeply; head side to the side; head up and down; bent over the wrist, jogging; talking; breath normally.

Each step took 60 s, and the Bitrex solution was refilled into the hood every 30 s, with half the dosage of the amount of the previous test. We asked the participant if he/she could taste the solution during each step of the test. The test was considered pass or fail.

We chose the qualitative fit test became it is more widely used [13], simpler to use, easy to transport [14], faster to perform, and cheaper to set up than the qualitative fit test.

Whenever the test failed, we would adjust the strap behind the respirator to tighten the face-seal leakage and repeat the procedure again. We would repeat the test twice before considering the test a failure.

Finally, we took note of the collected data, such as gender, age, size of the respirators, number of sprays, threshold level, and test result (pass, fail).

### Respirator performance

The real-time respirator performance test method was developed using a MT-05U machine (SIBATA model, Saitama, Japan), which measures particle concentrations of 0.03–0.06 μm diameter using a particle generator laser beam scattering particle counter, that measures particles outside and inside the mask. The ratio between these two values is considered the percentage leak, as follows:

$$\%leak=\frac{\left(Paticle\;inside\;the\;filter\right)}{\left(Paticle\;outside\;the\;filter\right)}x\;100$$

The percentage filter performance was calculated as 100 - % leak.

We tested at an airflow rate of 85 L/min, which is 3–4 times higher than normal physiology, assuming that there should be no leakage through the filter. We tested two types of filters, CareStar and SafeStar, that were incorporated into the silicone mask. CareStar is limited for 24-h usage, and SafeStar is for 72-h duration.

### Breathing resistance test (BR Test)

**Fig 4:**
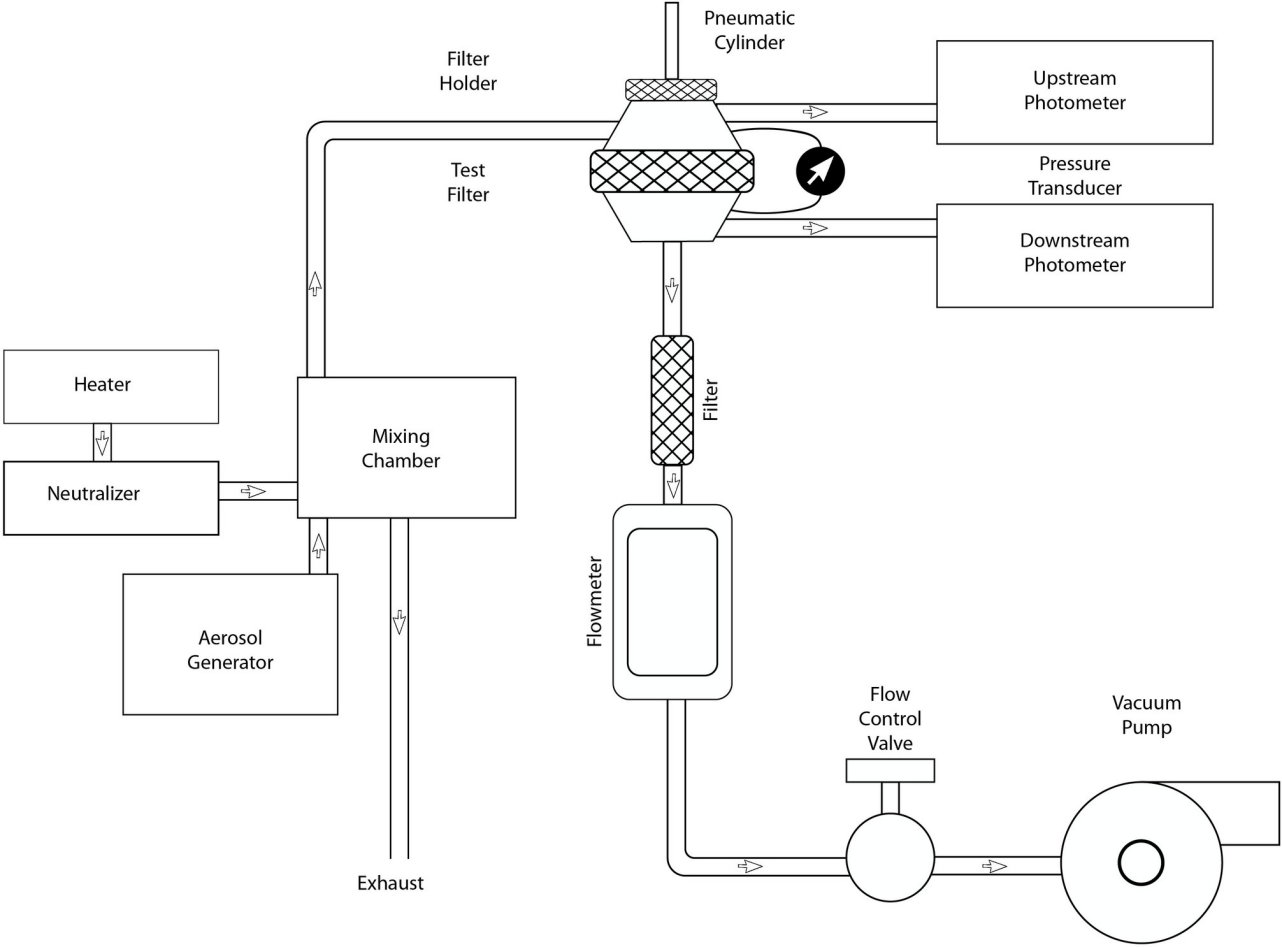
Filter Tester model 8130A. Ref: https://www.tsi.com/getmedia/05b5cb92-d6bb-4bb7-862f-59587ba52ffd/8127-8130_5001474_A4_WEB?ext =.pdf.

The inhalation BR Test was evaluated using TSI model 8130 Automated Filter Tester using the method described in the Code of Federal Regulations (Title 42 CFR Part 84) (NIOSH, 1995). NIOSH certifies FFRs when the inhalation BR is less than 35 mm of H_2_O and the exhalation BR is less than 25 mm of H_2_O measured at constant one-direction airflow of 85 L min-1 (NIOSH, 2005). Particle penetration was carried out in the chamber using a NaCl aerosol generated from a 2% solution applied to a nebulizer, as specified by NIOSH to certify N95 FFRs (42 CFR 84.181). The environmental conditions during both particle penetration tests (initial and final) were maintained at 38°C and 85% relative humidity. The test used two light-scattering laser photometers simultaneously to measure the upstream and downstream aerosol concentration levels at a constant flow that is reported with the TSI model 4045 flow meter. The particle penetration value is determined from the ratio of these two readings. A highly accurate electronic pressure transducer determines the filter resistance. Pressure transducer and photometer readings are taken between every test to determine the new pressure offset and photometer background values. Fig 4 provides a schematic of the experimental set-up that was used to measure BR through the respirator. First, an initial NaCl aerosol penetration test was performed following NIOSH certification protocol (42 CFR 84.181). Next, a BR test was performed.

### Study protocol

**Fig 5:**
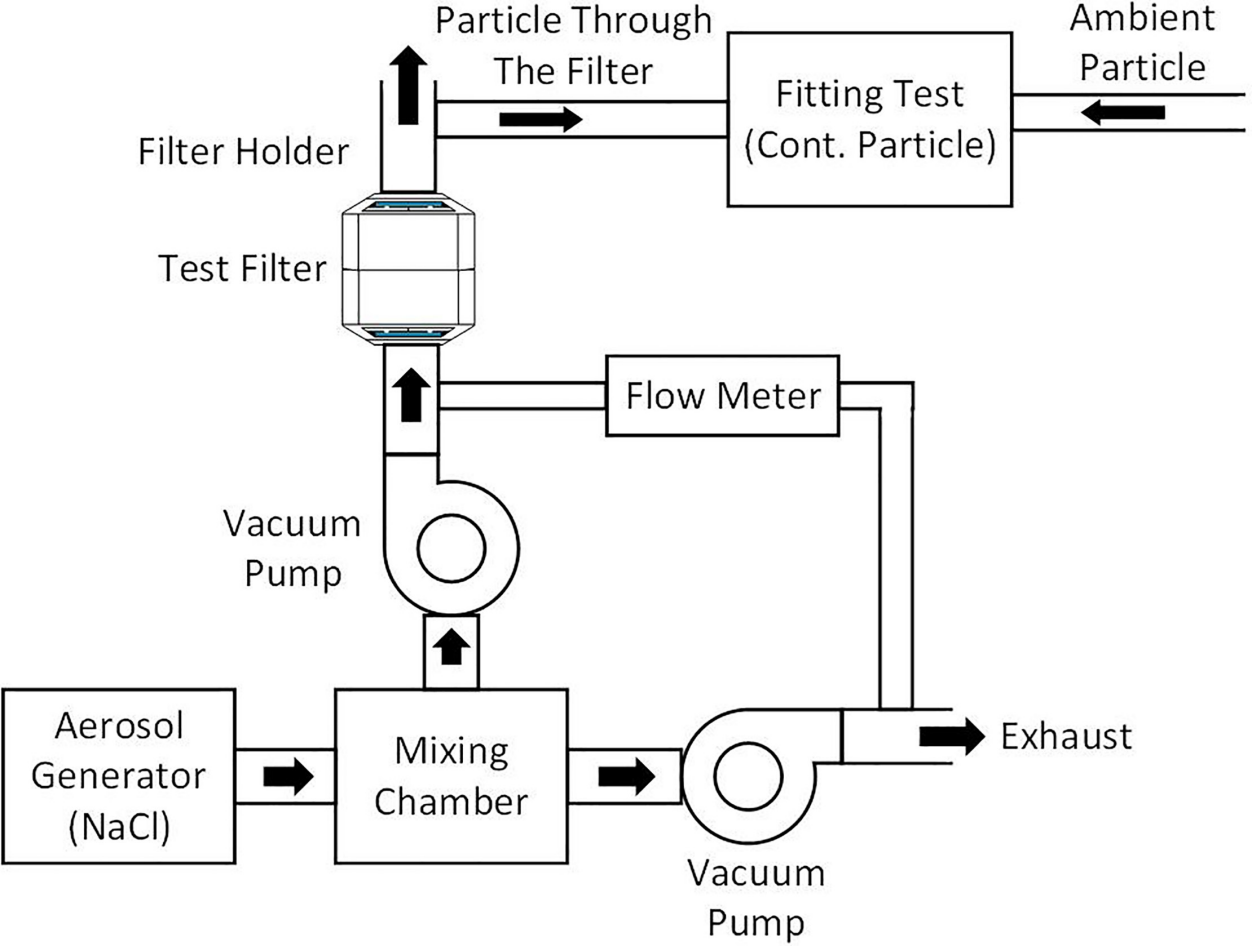
Diagram of the filtration test. dx.doi.org/10.17504/protocols.io.bnr2md8e.

We designed the filter test, as shown in Fig 5. We put the filter on the airflow generator, which generates an airflow of about 85 L/min. The NaCl aerosol condensation was generated by an MT-05 machine and flown through the cannula with a concentration of at least 70 particles/cc (the minimum level needed to conduct the test, in this study the average was 235 particles/cc) into the test chamber (green tube). We then measured the real-time percentage leak by counting the particles inside the filter compared to outside.

### Data collection

Sample size calculation.

### Fit test

We proposed the assumption that all subjects should pass the fit test by the non-inferiority hypothesis. The sample size estimation can be done by comparing the ratio of one group to the constant number (tooling for one population proportion) for non-inferiority trial as follows:

$$n=\frac{\left(Z_{1-\alpha }+Z_{1-\beta })p(1-p\right)}{{\left(p-po-\delta \right)}^{2}}$$

where *n* is the sample size, Z1−α2 is the statistical number under normal distribution according to ∝ = 0.05, and thus *Z*_1−*α*_ = 1.645, p is the ratio of sample size that passes the test at 99%, po is the reference value (100% Pan the test), and *δ* is the statistically significant value at 0.05.

$$Therefore:\;n=\frac{{\left(1.645+0.842\right)}^{2}\;x\;0.95(1-0.99)}{{\left(0.99-1.0-0.05\right)}^{2}}$$

n=39

We included at least 39 persons.

### Filter performance

We calculated the sample size using the test for non-inferiority for testing two dependent means as follows:

$$n=\frac{{{(Z}_{1-\alpha }+Z_{1-\beta })}^{2}\delta^{2}}{{\left(\Delta -\delta \right)}^{2}}$$

where Δ is the difference of the mean of the population, *δ* is the statistically significant value, and *δ*^2^ is the variance.

Since there are no previous studies to cite for references, we have used the G power version 3.1.9.4 program to compute filter performance. Two groups were compared using a paired t-test, where ∝ = 0.05 (one tall) and the power of the test is 80%. We applied effect size to 0.5 (medium effect size) [15].

n=27

We used the panel N99 respirators, which were used from 1 to 24 h to test for the filtration performance, relative to a control (new filter) respirator.

### Data analysis

The data analysis was performed using SPSS version 22.0 and Microsoft Office Excel for presenting the demographic data and respirator size. Descriptive statistics were calculated. The normal distribution of the data was tested using on the Shapiro-Wilk Test. The fittest passing rates (i.e., the number of subjects passing each fit test divided by the total number of subjects performing that fit test) were calculated. Filter penetration data were reported and calculated as mean ± SD, and compared to a specified target protection value of 99% in our respirator model.

## Results

Table 1 provides a summary of the baseline demographic of all participants. Forty-three subjects entered the study (22 male, 21 female). We excluded two persons due to test intolerance (fatigue during the test) and insensitive taste. The remaining 41 subjects (21 male, 20 female), mean age 28.22 ± 6.34 years old, used three sizes of the respirators: small (n = 10), medium (n = 25), large (n = 6) (Table 2).

**Table 1:** Baseline characteristics of participants performing fit test (n = 41).

Characteristics	
Gender, n (%)	
Male	21 (51.2)
Female	20 (48.8)
Age (year), mean±SD	28.22 ± 6.34
BW (kg), mean±SD	64.87 ± 20.10
BMI (kg/m^2^), mean±SD	23.20 ± 5.49
Number of tests, median (IQR)	10 (5–12)
(min—max)	(3–30)

**Table 2:** Distribution of mask sizes.

	Frequency	Percent	Valid percent	Cumulative percent
Valid S	10	24.4	24.4	24.4
M	25	61.0	61.0	85.4
L	6	14.6	14.6	100.0
Total	41	100.0	100.0	

Thirty-two subjects (78%) passed the first fit test. After adjusting the O-ring trap to tighten the respirators, seven subjects passed the second test (80%). There was one person who did not pass the test, even after adjusting the strap for the third time. The overall fit-test passing rate in this study was 40/41 (97.6%) (Table 3).

**Table 3:** Summary of the fit test results (N = 41).

	Result			
	Pass		Failed	
Fit test	n	Percent	n	Percent
1^st^ Fit (n = 41)	32	78.0	9	22.0
2^nd^ Fit (n = 9)	7	77.8	2	22.2
3^rd^ Fit (n = 5)	4	80.0	1	20.0
Cumulative result (n = 41)	40	97.6	1	2.4

### Filter penetration

**Fig 6:**
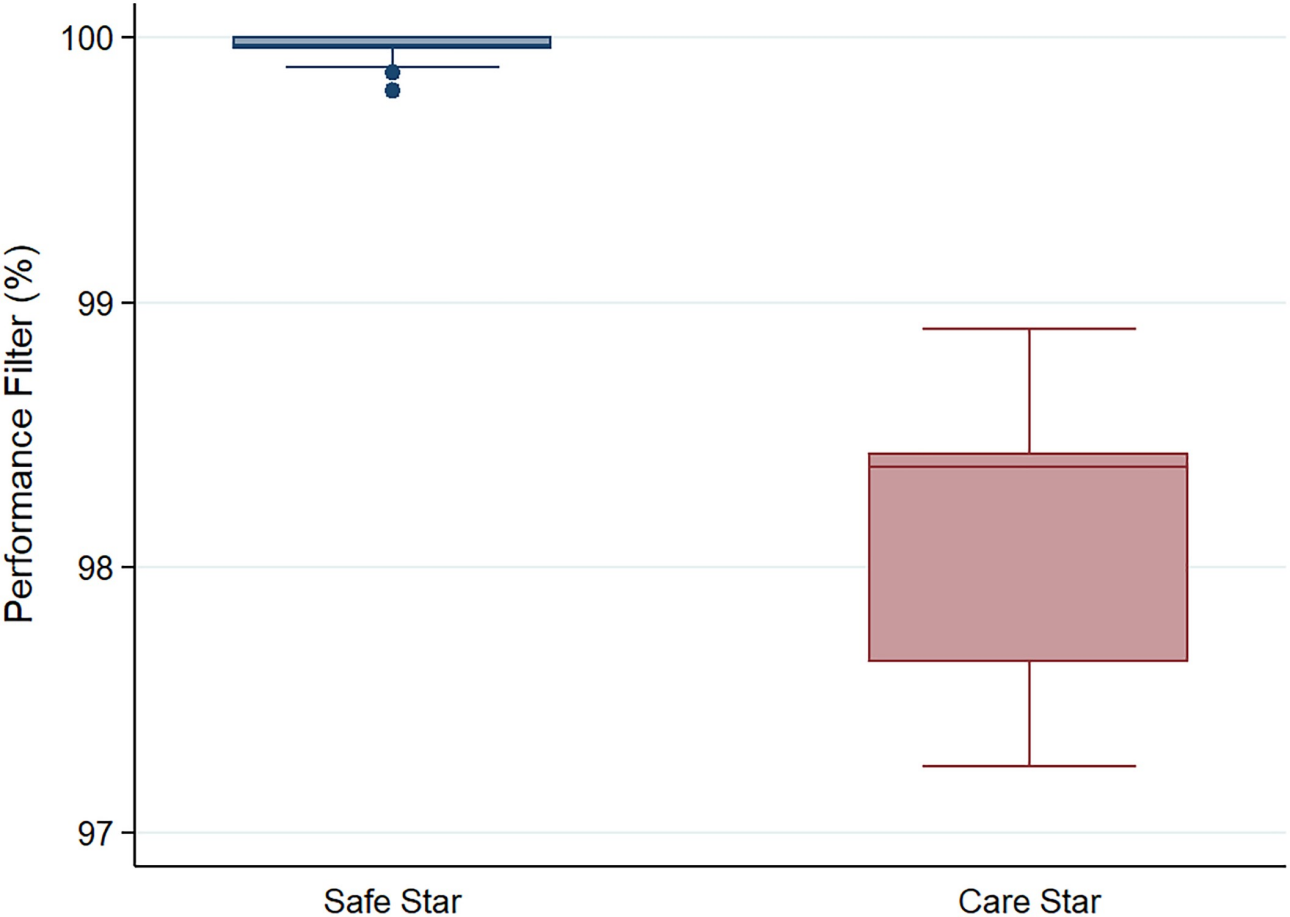
Comparison between the percentage performance of the SafeStar and CareStar filters.

Filler penetration for monodisperse aerosols in the 0.03-μm range aerosols was measured at constant flow rate of 85 ±40 L/min using the SIBATA MT 05U Fit test under the fit check mode (standard OSHA 29 CFR 1910.134). Table 4 shows the filler penetration of two filler types (SafeStar and CareStar, Draeger). We tested the baseline filter performance (first use) and used the filters for between 1 and 24 h. All of the filters had at least 99% protection (mean, 99.96 ± 0.06 for SafeStar, and 98.13 ± 0.56 for CareStar, p < 0.001). The SafeStar filter had better efficiency than CareStar (Table 5). Even with a use time of up to 24 h, the protection value remained > 99% (Fig 6).

**Table 4:** Data of percent filtration of each filter.

No.	Type	Outside	Inside	% Filter performance	Time of use (hr)	Amount	% Leak
1	Safe Star	7689	2	99.97	1	1	0.026
2	Safe Star	8374	2	99.98	1.3	1	0.023
3	Safe Star	8633	2	99.98	2	1	0.023
4	Safe Star	7681	2	99.97	2	1	0.026
5	Safe Star	8048	4	99.95	3	1	0.049
6	Safe Star	12871	17	99.87	3	1	0.132
7	Safe Star	7746	0	100	4	1	0
8	Safe Star	14718	4	99.97	5	1	0.027
9	Safe Star	5553	11	99.8	8	1	0.198
10	Safe Star	11676	4	99.97	8	1	0.034
11	Safe Star	7646	0	100	9	1	09
12	Safe Star	8523	3	99.96	10	1	0.035
13	Safe Star	12105	0	100	10	1	0
14	Safe Star	12422	14	99.89	24	1	0.112
15	CareStar	7986	129	98.38	2	1	1.615
16	CareStar	12092	190	98.43	4	1	1.57
17	CareStar	9199	101	98.9	10	1	1.09
18	CareStar	12754	200	98.4	10	1	1.56
19	CareStar	12356	14	97.64	24	1	0.113
20	CareStar	10651	213	97.25	1	1	0.08
21	Safe Star	8345	0	100	3	1	0
22	CareStar	2577	53	97.94	1	1	2.06
23	Safe Star	2694	0	100	30	1	0

**Table 5:** Mean filtration efficiency of the CareStar and SafeStar filters.

Variables	Total	SafeStar	CareStar	P-value
	23 (100)	16 (69.57)	7 (30.43)	
Particle outside, Mean±SD	9232.13±3129.90	9045.25±3019.50	9659.29±3580.54	
(Min-Max)	(2577–14718)	(2694–14718)	(2577–12754)	
Inside, Mean±SD	41.96±71.19	4.06±5.26	128.57±77.03	
(Min-Max)	(0–213)	(0–17)	(14–213)	
% Performance Filter, Mean±SD	99.40±0.91	99.96±0.06	98.13±0.56	<0.001
(Min-Max)	(97.25–100)	(99.80–100)	(97.25–98.90)	

### Breathing resistance test

As shown in Table 6, The mean penetration values for the medium size respirators were below the NIOSH certification limit. However, the mean penetration values obtained for small and large respirators exceeded the 5% NIOSH certification limits. The mean penetration value for small size respirators was 95% and 98.65% for the large size respirators. None of the respirators exceeded the NIOSH certification limit for BR of 35 mm H_2_O [16].

**Table 6:** Breathing resistance test of half-piece respirator.

Model	Standard		Pre-conditioning	Particulate Loading Tests				Air Perm & Resistance	
	Org	Name	Pre-conditioning summary	Test 1	Test 2	Loading Procedure	Minimum Efficiency	low Rate	Maximum Resistance
1 VJR-NMU (S)	NIOSH42 cfr part 84	N95	38⁰C, 85% humidity, 25 hrs	NaCl .26 μm	-	Loaded until 200 mg	95.00	84.86	0.51
2 VJR-NMU (M)	NIOSH42 cfr part 84	N95	38⁰C, 85% humidity, 25 hrs	NaCl .26 μm	-	Loaded until 200 mg	87.59	84.87	0.93
3 VJR-NMU (L)			38⁰C, 85% humidity, 25 hrs	NaCl .26 μm	-	Loaded until 200 mg	98.65	84.85	3.27

## Discussion

It is widely accepted that wearing face masks in public corresponds can help to prevent inter-human transmission of SARs-CoV2 [17]. In HCP that remain at risk to COVID-19, patients should follow appropriate infection control procedures. These levels of protection depend on the setting where modes of viral transmission are relevant. Filtering facepiece respirators are essential devices to protect HCW from bioaerosol particles [18, 19]. According to the National Institute for Occupational Safety and Health (NIOSH) regulations 42 CFR 84, N 95 respirators are recommended for personal protection from exposure to respiratory aerosol particles [20]. However, due to the COVID-19 pandemic, there is a global supply shortage for the most exposed persons, HCWs. This underlines the urgent need to replenish the masks using a variety of available materials.

Our team has invented a novel elastometric half-piece respirator by combining a silicone facemask (as used in the operating rooms) with an electrostatic filter plug (CareStar or SafeStar Draeger Lubeck, Germany). We developed an O-ring strap made from silicone, similar to the silicone mask, to tighten the mask to the face. The strap consists of three parts: the silicone strap; the strap locker to adjust the length (made from polypropylene); and a 4-way hook (made from silicone to connect the strap with the mask).

By adjusting the O- ring straps, almost all patients passed the fit test. This shows a good level of protection. Adjustable head straps then allow a better-customized seal because they can be tightened to better secure the respirators. At first, three different sizes of the face pieces were produced. The majority of our participants used medium sizes. It should be noted that fit-testing is just one factor to determine the level of protection provided by a respirator. Other tests, such as filter penetration, should also be done. According to the specification of the electrostatic filter (CareStar or SafeStar), the estimated filtration efficiency should be more than 99% after testing with the SIBATA machine. The new filter had the filtration efficiency of more than 99%. We then evaluated the filters after use (1 to 24 h) since the filter efficacy might be hampered by humidity and the formation of a biofilm layer over the filter surface. The test protocol that we adapted measures the percent leakage through the filters by counting the sodium chloride generated aerosols. The percent of leakage was less than 1%. Thus, we can confirm that the efficiency of our new respirators was compatible with the N99 type of respirator and can be used at least up to 24 h before changing the filter.

### Limitations

The limitations of this study were the method of fit test, that was qualitative and should be confirmed with qualitative fit test. The silicone mask still has some drawbacks, such as the hardness of the material, that is comfortable for the wearer. Those who wear eyeglasses might have difficulty placing his/her eyeglasses on the bridge of the silicone mask. The levels of protection afforded by the respiratory protective devices in this study might not be representative of all respirator wearers, who might have different facial size distributions than those of the subjects in this study. The filters that we tested had limited use times (up to 24 h), while their efficiency remained excellent. Further tests should be performed for a filter that has been used for more than 24 h. The filtration test was designed with the equipment available in the unit based on the urgency to search for alternative protective devices. We found that 42.6% of the wearers reported some discomfort due to the haziness and fog inside the silicone mask, which was caused by the condensation of the humidity inside the mask. The hydrophobicity of the silicone, which protects water and droplets to penetrate, promoted the discomfort. There were also some hearing problems while wearing the silicone mask as found in the Powered Air Purifying Respirator, PAPR) since the mask decreases the vibration of noise causing communication errors.

**Fig 7:**
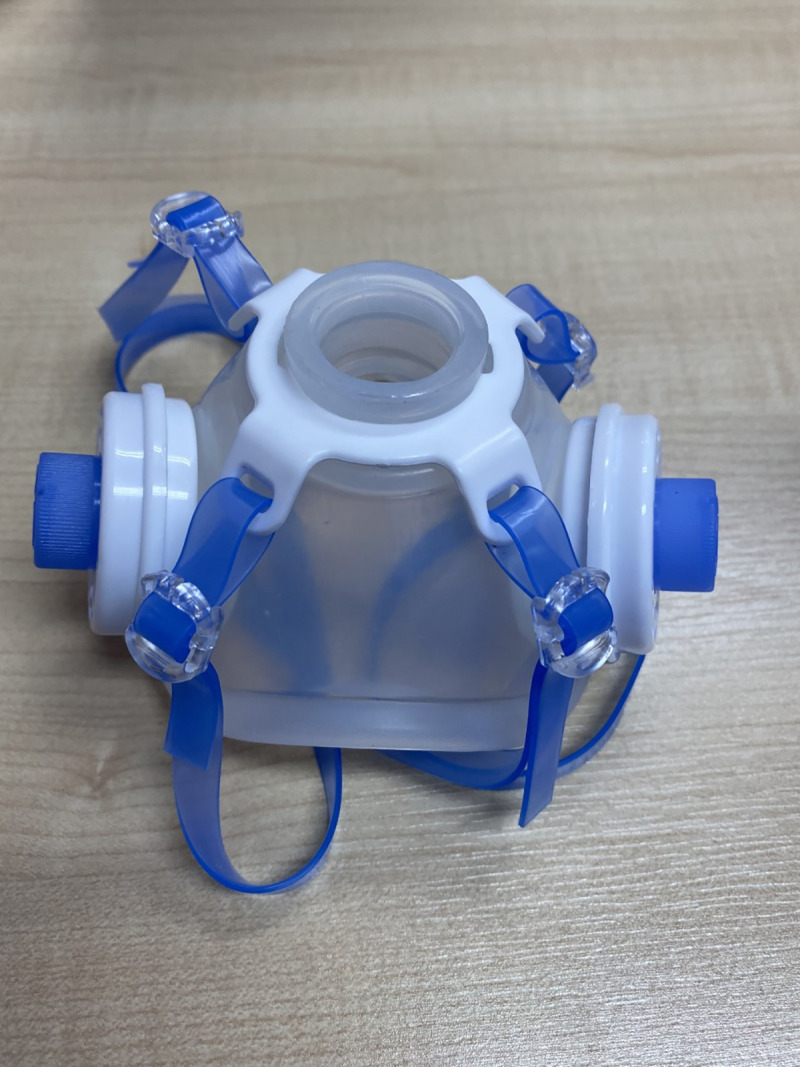
New version of silicone mask with exhalation valve.

The silicone mask did not have an exhalation valve, carbon dioxide accumulation within the mask is inevitable. The maximum time to wear in our group was 4 h. The wearers needed to doff the respirator and donned again after the rest period. We plan to invent a new generation of half-piece respirator with an exhalation valve which can be adjustable on and off manually depending on the purpose of the wearer. The result will be soon be reported. (Fig 7).

Despite the limitations of our study, a strength of this innovation is that the materials are available, and the efficacy is acceptable, and even superior to the long-used N95 masks.

## Conclusions

Here we report the efficacy of our innovation, a silicone N99 mask that we have named VJR-NMU N99 half-piece respirators. The VJR-NMU N99 respirators surpassed the expected levels of protection, and can be useful in the context of a global shortage of PPE. However, this is the first version of our masks, and further modifications are needed to improve user friendliness and provide adequate protection. We believe that the findings of this study will contribute to the provision of safe and superior healthcare services for HCW, and that the VJR-NMU N99 respirators can help to replenish the shortage of essential healthcare worker protection.

## Supporting information

S1 File (DOCX)

## References

[1] World Health Organization. Rational use of personal protective equipment for coronavirus disease (COVID-19) and considerations during severe shortages 2020. Available from: file:///C:/Users/User/Downloads/WHO-2019-nCov-IPC_PPE_use-2020.3-eng%20(1).pdf.

[2] Gandhi R, Lynch J, Rio C. Mild or moderate Covid-19. N Engl J Med. 2020 10.1056/NEJMcp2009249 32329974

[3] Gralton J, Tovey E, Mclaws ML, Rawlinson WD. The role of particle size in aerosolized pathogen transmission: A review. J Infect. 2011;62: 1–13. 10.1016/j.jinf.2010.11.010 21094184 PMC7112663

[4] Leung NHL, Chu DKW, Shiu EYC, et al Respiratory virus shedding in exhaled breath and efficacy of face masks. Nat Med. 2020;26: 676–680. 10.1038/s41591-020-0843-2 32371934 PMC8238571

[5] Lepelletier D, Grandbastien B, Romano-Bertrand S, Aho S, Chidiac C, Géhanno JF, et al French Society for Hospital Hygiene and the High Council for Public Health. What face mask for what use in the context of COVID-19 pandemic? The French guidelines. J Hosp Infect. 2020 4 26;105(3):414–8. 10.1016/j.jhin.2020.04.036 Epub ahead of print. ; PMCID: PMC7194956. 32348833 PMC7194956

[6] World Health Organization (WHO). Coronavirus disease (COVID-19) advice for the public: When and how to use masks. October 2020. Available from: https://www.who.int/emergencies/diseases/novel-coronavirus-2019/advice-for-public/when-and-how-to-use-masks.

[7] Occupational Safety and Health Administration (OSHA): 29 CFR parts 1910 and 1926 Respiratory Protection: Final Rule. Federal Register 63(5): 1278–1279. Washington, D.C: U. S. Government Printing Office, Office of the Federal Register, 1 8,1998.

[8] Centers for Disease Control and Prevention: Laboratory performance evaluation of N95 filtering facepiece respitators;1996. MMWR 47:1045–1049(1998). 9869077

[9] Zhuang Z, Bradtmiller B, and Shaffer RE [2007]. New respirator fit test panels representing the current U.S. civilian workforce. Journal of Occupational and Environmental Hygiene 4: 647–659. 10.1080/15459620701497538 17613722

[10] OSHA, Title 29 CFR.1910.134. Respiratory Protection Program Standards- Fit Testing Procedures (Mandatory) Available from: Washington, Occupational Safety & Health Administration (OSHA), Government Publishing Office. [Standard], 2016. Available form: https://www.osha.gov/pls/oshaweb/owadisp.show_document?p_table=STANDARDS&p_id=9780.

[11] Moldex–Safety Data Sheets (SDS) of Bitrex Sensitivity and Fit Test Solutions. Available from: https://www.moldex.com/resources/important-information/material-safety-data-sheets-msds/.

[12] Coffey C, Zhuang Z, Campbell D. Evaluation of the Bitrex qualitative fit test method using N95 filtering-facepiece respirator. J Int Soc Respir Protection. 1998;16: 48–54.

[13] Milchell B, McGregor W, McKenzie D. Can homemade fit testing solutions be as effective as commercial products? Healthc Infect. 2012;17: 111–114. 10.1071/HI12019 32288839 PMC7129124

[14] Mullins HE, Danisch SG, Johnston AR. Development of a new qualitative test for fit testing respirators. Am Ind Hyg Assoc J. 1995;56: 1068–1073. 10.1080/15428119591016278 7502992

[15] Cohen J. Statistical power analysis for the behavioral sciences. Hillsdale, NJ: Lawrence Erlbaum Associates Inc; 1917.

[16] Centers for Disease Control and Prevention. The National Personel Protective Technology Laboratory. (NPPTL). In:CDC Web Achive. available from: https://www.cdc.gov/niosh/npptl/topics/respirators/pt84abs2.html

[17] Zhang R, Li Y, Zhang AL, Wang Y, Molina MJ. Identifying airborne transmission as the dominant route for the spread of COVID-19. PNAS. 2020;117: 14857–14863. 10.1073/pnas.2009637117 32527856 PMC7334447

[18] Loeb M, Dafoe N, Mahony J, John M, Sarabia A, Glavin V, et al Surgical mask vs N95 respirator for preventing influenza among health care workers: a randomized trial. JAMA. 2009;302: 1865–1871. 10.1001/jama.2009.1466 19797474

[19] Radonovich LJ Jr., Simberkoff MS, Bessesen MT, Brown AC, Cummings DAT, Gaydos CA, et al N95 Respirators vs medical masks for preventing influenza among health care personnel: a randomized clinical trial. JAMA. 2019;322: 824–833. 10.1001/jama.2019.11645 31479137 PMC6724169

[20] National Institute for Occupational Safety and Health (NIOSH). NIOSH respirator selection logic 2004. DHHS (NIOSH) publication no. 2005–100. Cincinnati, Ohio: NIOSH; 2004.

