# Supplementary material for: The new silicone elastometric half-piece respirator, VJR-NMU: A novel and effective tool to prevent COVID-19
Source: PLoS One. 2020 Dec 31;15(12):e0237206. doi: 10.1371/journal.pone.0237206 (PMC7774960; doi:10.1371/journal.pone.0237206)
Supplement: S1 File — (DOCX) [file pone.0237206.s001.docx]

**Data of Face Dimension in our Study Group**

| **TABLE1.** Face Length and Face Width | |
| --- | --- |
| **Variable** |  |
| Face width (mm), mean ± SD | 142.20 ± 22.07 |
| (min - max) | (110 - 225) |
| Face length (mm), mean ± SD | 118.45 ± 14.03 |
| (min - max) | (88 - 150) |
| Lip length (mm), mean ± SD | 64.5 ±13.38 |
| (min - max) | (42 - 95) |

| **TABLE2.** Percentage of Population and Number of Subjects for the Panel Based on Face Length and Face Width | | | | | | | | | | | |
| --- | --- | --- | --- | --- | --- | --- | --- | --- | --- | --- | --- |
| **Cell** | **Male** | | **Female** | | **Total** | |  |  |  |  |  |
|  | **n** | **(%)** | **n** | **(%)** | **n** | **(%)** |  |  |  |  |  |
| 1 | 1 | (4.5) | 2 | (10.5) | 3 | (7.5) |  |  |  |  |  |
| 2 | 0 | (0.0) | 0 | (0.0) | 0 | (0.0) |  |  |  |  |  |
| 3 | 2 | (9.5) | 1 | (5.3) | 3 | (7.5) |  |  |  |  |  |
| 4 | 0 | (0.0) | 1 | (5.3) | 1 | (2.5) |  |  |  |  |  |
| 5 | 0 | (0.0) | 6 | (31.6) | 6 | (15.0) |  |  |  |  |  |
| 6 | 2 | (9.5) | 1 | (5.3) | 3 | (7.5) |  |  |  |  |  |
| 7 | 3 | (14.3) | 0 | (0.0) | 3 | (7.5) |  |  |  |  |  |
| 8 | 0 | (0.0) | 1 | (5.3) | 1 | (2.5) |  |  |  |  |  |
| 9 | 3 | (14.3) | 0 | (0.0) | 3 | (7.5) |  |  |  |  |  |
| 10 | 3 | (14.3) | 0 | (0.0) | 3 | (7.5) |  |  |  |  |  |
| Total | 12 | (63.2) | 14 | (66.7) | 26 | (65.0) |  |  |  |  |  |


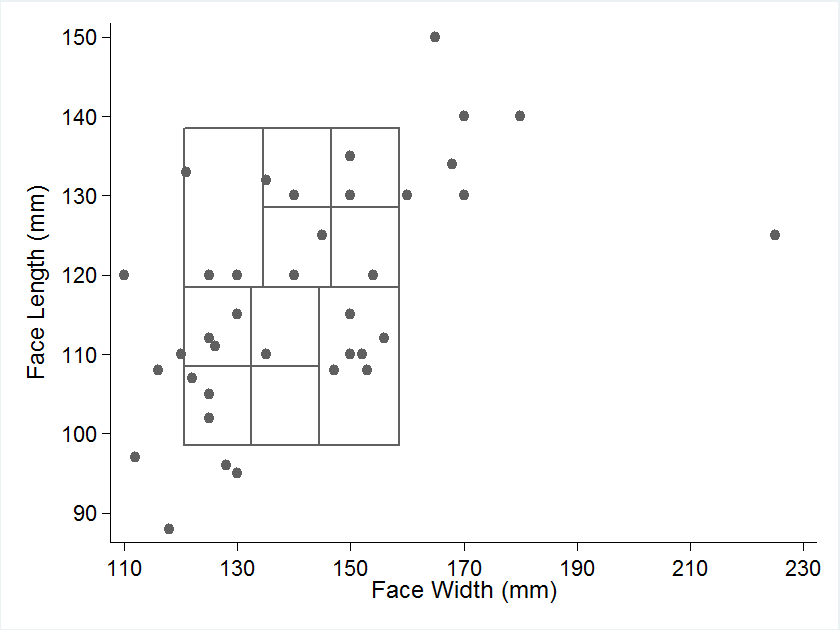


Figure 1 : The Scatter Plot of the Bivariate Distribution of the Subjects

65% of the subjects are within the boundary of the panel.
